# Drug Interactions of Chronic Neuropsychiatric Drugs in Neuro-critical Care

**DOI:** 10.5005/jp-journals-10071-23195

**Published:** 2019-06

**Authors:** Shobhana A

**Affiliations:** Department of Neurocritical Care and Stroke Medicine, Institute of Neuroscinces, Kolkata, West Bengal, India

**Keywords:** Adverse drug reactions, Chronic, Neuropsychiatric, Neuro-critical care, Potential drug-drug interaction (pDDIs)

## Abstract

Neuro psychiatric illnesses are commonly recognised these days in the intensive care especially with the increasing aging population and more intensive care admissions. However they are still inadequately diagnosed and treated disease entities as a majority of these patients do not seek the help of specialists psychiatrists Of course the number of drugs used in psychiatry has explosively increased in recent years. As a corollary to this, the phenomenon of drug- drug interaction between psychiatric drugs and other drugs has come to the forefront. Drug- drug interaction (DDI)) is the response (pharmacological or clinical) of altered drug effects or increase in adverse effects when two or more drugs are used simultaneously^1,2^ This effect may be different from the usual action of the individual drugs when used alone. Potential drug- drug interaction (PDDI) are those where theoretically there may be an interaction between the drugs but have not clinically occurred.^1,2^

**How to cite this article:**

Shobhana A. Drug Interactions of Chronic Neuropsychiatric Drugs in Neuro-critical Care. Indian J Crit Care Med 2019;23(Suppl 2):S157–S161.

## INTRODUCTION

Neuro psychiatric illnesses are commonly recognised these days in the intensive care especially with the increasing aging population and more intensive care admissions. However they are still inadequately diagnosed and treated disease entities as a majority of these patients do not seek the help of specialists psychiatrists Of course the number of drugs used in psychiatry has explosively increased in recent years. As a corollary to this, the phenomenon of drug- drug interaction between psychiatric drugs and other drugs has come to the forefront.

Drug- drug interaction (DDI)) is the response (pharmacological or clinical) of altered drug effects or increase in adverse effects when two or more drugs are used simultaneously.^1,2^ This effect may be different from the usual action of the individual drugs when used alone. Potential drug-drug interaction (PDDI) are those where theoretically there may be an interaction between the drugs but have not clinically occurred.^1,2^

## EFFECTS OF DDI

DDIs are often the commonest cause of adverse drug reactions leading to increase in organ damages, morbidity, ineffective therapy, increased length of hospital stay, expenses as well as poor long term outcome and mortality.^1,2,3,4^ The adverse reactions often affect the cardiovascular and nervous systems.

### The Vulnerable Population

The elderly and the critically ill patients are more at risk of adverse events of DDIs as they are the groups where polypharmacy is common. The pharmacokinetics of the medications involved along with the compromised organ systems adds to the often higher doses and longer duration of drug administration in the intensive care unit (ICU). Most of the drugs used are parenteral formulations where the adjuvants added may increase the toxicity of the drugs.^4,5,6^

### Prevalence and Factors Determining DDI in ICUs

The prevalence of drug interactions in ICU, in general, ranges from 45% to 85%.^1,2^ Determinants of drug interactions depend upon number of drugs (increase in incidence of interactions with increased number of drugs), age, type of drug, presence of organ damage or comorbidities. Incidence of PDDI increases by 10-20% in patients using 10–20 drugs.^6^ In one study the average number of drugs used in an ICU patient was found to be seven^6,7^ and about 45% of the ICU prescriptions included some PDDI.

### Drugs Commonly Involved in DDIs

In multispecialty ICUs, dexamethasone, frusemide, nifedipine and enoxaparin were identified to have highest frequency of PDDI in some studies.^7^ In neurocritical care units anticonvulsants and psychiatric medications often are involved in DDIs. The most frequent interactions are between drugs acting on the cardiovascular system, corticosteroids, antibiotics, antidepressants, antipsychotics, immunosuppressants and opioids.^4^ Nervous system medications account for 40% of DDI and midazolam and fentanyl were most frequently associated with DDI.^4^

### Mechanism of DDIs

Pharmacokinetic types of DDI are common and involve the absorption, metabolism, distribution and elimination of drugs. Usually PDDIs are often between drugs metabolized by the same Cytochrome P450 (CYP 450) enzymes and/ or due to concomitant administration of drugs that are inducers or inhibitors of these enzyme systems.^1^ Drugs metabolized by this route include drugs like midazolam, tacrolimus, cyclosporine and phenytoin. CYP450 inducers and inhibitors of clinical importance commonly used in ICU are amiodarone, fluconazole and carbamazepine (CBZ). Half of all drugs are metabolized by the CYP450 system and most DDIs involve the first - pass metabolim. Enzyme systems include CYPIA2, CYP2B6, CYP2C9, CYP2C19, CYP2D6, CYP2E1 and CYP3A4. Of mention here is the P glycoprotein transporter system (P-gp) which is found in the gastrointestinal and central nervous system and which determines the transport of drugs across the blood brain barrier. Most drugs used in psychiatry use this system. Pharmacodynamic interaction may also be seen and involves the change in the mechanism of drug action. Genetic polymorphism may also alter drug action.

**Table 1:** Commonly used psychiatric medications and adverse effects due to drug interactions

*Drugs*	*Adverse effects*
Haloperidol First gen. antipsychotics (especially when combined with amiodarone, levofloxacin) Atypical antipsychotics Lithium in overdose	QT prolongation Ventricular arrhythmias Torsades de Pointes(TdP)
SSRI, SNRI, when combined with linezolide, meperidine, tramadol, metoclopramide, ondansetron.	Serotonin syndrome
Serotonin inhibitors with antiplatelets or anticoagulants	Bleeding risk
Quetiapine	Hyperglycemia
Phenothiazines especially chlorpromazine, SSRI, TCA, bupropion, clozapine, haloperidol, lithium.	seizures
SSRI with statin	Vasospasm after SAH
SSRI, SNRI, TCA	Drug fever
Antipsychotics (typical > atypical) when combined with metoclopramide, or simultaneous withdrawal of dopaminergic drugs like levodopa.	Neuroleptic malignant syndrome

*Note*: Fluphenazine, risperidone have moderate risk of QTc prolongation and TdP while olanzapine and quetiapine have low risk. Arpiprazole has the least risk. Sertraline is relatively safe in cardiac patients

**Table 2:** Effects of DDIs between psychiatric drugs and other ICU drugs

*Drug class*	*Interaction with*	*Effect*
Antidepressants	Bupropion, Fluoxetine, Paroxetine	↑TCA levels
TCA	St John's Wort	↓Antidepressant efficacy
SSRI	Bupropion	↑Conc of SSRI
Sertraline, fluvoxamine	S-Warfarin	↑Bleeding
Antipsychotics Clozapine Olanzapine Haloperidol	Smoking	↓Level of antipsychotics
Haloperidol	Opioids	↑Opioid level
Sedaive Hypnotics	Carbamazepine(CBZ),Glucocorticoids, Phenytoin, Rifampicin, St John's Wort(all enzyme inducers)	↓Sedative effects ↑Anxiety
	Cimetidine, Ketoconazole, few Antiretrovirals & antibiotics inhibit enzymes	↑Sedation
Drugs for Attention Deficit	Dexamethasone, Rifampicin (enzyme inducers)	↓Effect of ADHD medicines
Hyperkinetic Disorder (ADHD)	H1 & H2antagonists, certain antidepressants are inhibitors	↑ADHD levels
Bipolar Medications Valproic acid	CBZ induces many medicines Warfarn	↓Effect ↑Bleeding
Lithium	ACE inhibitors, ARB, NSAID, diuretics	↓Renal excretion, ↑Li level
Drugs for EPS Effects Bromocriptine	Inhibits metabolism of many drugs	↑Drug levels
propranolol	Inhibits metabolism of many antipsychotics	↑Drug levels

## CLASSIFICATION OF PDDIS

It is advantageous to be aware of PDDIs. This helps clinicians to be cautious while prescribing drugs of different classes. Various softwares and databases have been developed to identify DDIs and PDDIs.^1,4^ Yet some may still be undiscovered. PDDIs are classified as:

**Table 3:** Illustrations of DDIs

*Drug class*	*Interaction with*	*Effect*
Antidepressants	Antiplatelets, Anticoagulants	↑bleeding
Agents which increase serotonin concentration	Entacapone	Serotonin syndrome, ↑nor epinephrine, dopamine, serotonin, ↑blood pressure
	Ergotamine and Sumatryptan which increase serotonin and nor epinephrine	Serrotonin syndrome
MAOI	Linezolide	Serotonin-syndrome, hypertensive crisis
Antipsychotics Dopamine antagonists	Levodopa, Pramipexole, Amphetamines, Methylphenidate Amantadine	↓Antipsychotic effect
Aripiprazole (dopamine agonist)	Metoclopromide, antipsychotics	EPS, ↓other antipsychotics
Sedative-hypnotics	Alcohol, Amphetamines, Anti psychotics	↑Sedative effect
BZD	GABA agonists	↑Sedative effect
ADHD Medications SSRI, TCA, Duloxetine, Venlafaxine, Bupropion	ADHD medicine	↑Serotonin Syndrome, ↑Blood pressure, ↑heart rate
Bipolar Disorder Medications Valproic acid	Lamotrigine	↑Lamotrigine level, chance of Steven Johnson
EPS Medication	Antipsychotics, atropine	↑anticholinergic effect

- Contraindicated for concurrent use.
- Major- Life threatening adverse events that may necessitate intervention and if these drugs are used the clinician needs to justify the usage of the same.
- Moderate- Most PDDIs fall in this category. These may exacerbate patients’ illness and warrant alternate therapy.
- Minor- Usually do not produce adverse events but in the eventuality of one, needs change in the treatment strategy.

### Psychiatric Illness and Critical Care

Patients with psychiatric diagnoses are common among severely ill patients and may represent a significant proportion of those treated in the ICUs.^8^ Often patients on psychotropic medications admitted to the ICU may require dose reduction or discontinuation of these drugs and this may lead to severe complications and even acute psychosis.^9^ Withdrawal after long term intake of baclofen has found to cause delirium, agitation and seizures. Withdrawal of benzodiazepines may precipitate seizures, agitation and hallucinations while that of SSRIs leads to flu-like illness. Many critically ill people suffering from COPD, diabetes or cardiovascular disorders suffer from mental illness and this leads to increased morbidity Chronic psychiatric medication or associated substance abuse could induce immunosuppression as suggested in several studies and could worsen organ dysfunction and increase risk of nosocomial infections in these population during ICU stay.^10^ On the other hand 60–80% of critically ill patients especially on mechanical ventilation may suffer from pain, agitation, insomnia and delirium and are regularly given analgesics, sedatives and antipsychotics. These add to the morbidity due to DDIs and adverse reactions.^11^ It is implied that a thorough history of prior health status including medication intake should be taken in all. About 3% of the patients requiring mechanical ventilation had one or more psychiatric illness in a Danish study.^12^ Majority did not have any psychiatric illness or medication prior to hospitalization in ICU. Mood and anxiety disorders after a critical illness were common. The primary psychiatric disorders admitted to ICU include anxiety disorders, schizophrenia, depression, bipolar disorders, depression, substance abuse or dependence.^13^ Self- harm including drug overdosages, hanging, drowning, corrosive ingestion, organophosphorus poisoning are common.^11^ Hence patients with psychiatric disorders may be admitted with critical illness or critically ill patients may develop psychiatric disorders while in ICU.^11^

### Use of Psychiatric Drugs in ICU

Benzodiazepines (BZD), antidepressants and antipsychotics are often the first psychiatric medicines to be added in the critical care units.^13^ BZD (midazolam and lorazepam have been used to manage agitation in the ICU for ages However the current recommendations suggest a non BZD agent for sedation in ICU.^13^ The antipsychotics used include the first generation ones with high affinity for dopaminergic (D2) receptors while second generation agents are the serotonin receptor antagonists (5HT2), used in schizophrenia, agitation and delirium. Nowadays atypical antipsychotics like quetiapine, olanzapine and risperidone are being more frequently used due to less side effects. Haloperidol which is an age old drug for delirium may precipitate alarming extrapyramidal symptoms (EPS).^13^ First generation antipsychotics like thioridazine and chlorpromazine which are useful in hyperactive delirium have more anticholinergic effects and may produce arrhythmias as well as EPS (D2 inhibition).To summarise, BZDs, neuroleptics, antipsychotics other than neuroleptics, lithium, selective serotonin reuptake inhibitors (SSRIs), serotonin -norepinephrine reuptake inhibitors (SNRIs), tri and tetra cyclic antidepressants (TCA) and monoamine oxidase inhibitors (MAOI) are commonly used in psychiatric patients.^11^

### Adverse Effects of Psychiatric Drugs

Most DDIs exacerbate the known side affects of the interacting drugs. Hence it is important to be aware of the common adverse effects of psychiatric drugs before knowing the drugs implicated in interaction (Table 1).^13-15^

Below is a list of common pharmacokinetic and pharmacodynamics interactions of psychiatric drugs with others maily cardiovascular drugs (Tables 2 to 5)^13-16^.

**Table 4:** Pharmacokinetic interactions of psychiatric medications and other drugs

*Cardiac drug*	*Psychiatric drug*	*Effect*
Bretylium	Amphetamine, Methylphenydate, MAOI	↓effects of Bretylium
Bretylium	TCA	Interferes with Bretylium action
Clonidine	B Blocker	Severe hypotension
Clonidine	Calcium Channel Blocker (Non-Dihydropyridine)	Hypotension, AV block
Clonidine	TCA	Hypotension
Frusemide	Fluoxetine	Hyponatremia
Ibutilide	Haloperidol, Phenothiazines, TCA	↑QTc
Quinidine	TCA	↑QTc
Dobutamine, Dopamine, Phenylephrine	MAOI, TCA	↑Blood Pressure(BP), arrythmias

**Table 5:** Pharmacodynamics interactions of psychiatric drugs and other drugs

ACE Inhibitor	TCA	↑TCA, confusion, insomnia, mood changes
Atropine	Amantadine	Anticholinergic side effects
Atropine	Haloperidol	↓ Haloperidol levels, tardive dyskinesia
Atropine	Phenothiazine	↑ Anticholinergic effects, ↓ phenothiazine effects
Propranolol	Chlorpromazine, Thioridazine	Each inhibits metabolism of other
Propranolol	SSRI	↑ B Blocker
Calcium Channel Blocker (Non-Dihydropyridine)	BZD	↓ BZD metabolim
Calcium Channel Blocker	CBZ	↓ Calcium Channel Blocker (CCB) level CCB ↑ CBZ levels
CCB(Non- Dihydropyridine)	Lithium	↓ or ↑ Lithium
Clonidine	Phenothiazines	↓ BP
Digoxin	BZD	May ↑ Digoxin level
Hydrochlorothiazide	CBZ	Hyponatremia
Nimodipine	CBZ	↓ Nimodipine level
Nimodipine	Valproic acid	↑ Nimodipine level
Warfarin	CBZ	↓ PT, INR
Warfarin	SSRI	↑ Warfarin effect
Warfarin	Paroxetin	↑ Bleeding
Warfarin	Trazodone	↓ PT, INR

Neurocritical care units often face the complex situations where patients with cardiac disorders co- exist with neurological and psychiatric diseases. As has been mentioned earlier DDIs with cardiac medications or cardiovascular adverse effects of drug combinations may precipitate life threatening situations in the ICU. Following is a list of some important DDIs between cardiovascular and psychotropic drugs.^16^ The major DDIs are mentioned in Table 4 and the moderate ones in Table 5.

## CONCLUSION

Neurocritical care units deal with unique and complex disease states involving often multiple organ systems and poly pharmacy is unavoidable. Psychotropic drug usage for primary psychiatric illness or for psychiatric symptoms developing after ICU admission is increasing Drug- drug interactions is a natural fall out in this situation. It is important to be prepared for such situations. Softwares and databases containing these information are easily accessible. Documenting such adverse events should be a part of the hospital protocol.

## References

[1] Rodrigues AT,, Stahlschmidt R, (2017;). Prevalence of Potential Drug-Drug Interactions. Braz. J. Pharm. Sci..

[2] Shakeel F,, Aamir M, (2018;). Epidemiology of Potential Drug-Drug Interactions in Elderly Population Admitted to Critical Care Units of Peshawar, Pakistan.. BMC Pharmacology and Toxicology..

[3] Baniasadi S, (2015;). Important Drug Classes Associated with Potential Drug–Drug Interactions in Critically Ill Patients: Highlights for Cardiothoracic Intensivists. Ann.. Intensive Care..

[4] Smithburger PL,, Sandra L, (2012;). Drug–Drug Interactions in the Medical Intensive Care Unit: An Assessment of Frequency, Severity and the Medications involved.. International Journal of Pharmacy Practice.

[5] Devlin JW,, Mallow-Corbett S,, Riker RR. (2010;). Adverse drug events associated with the use of analgesics, sedatives, and antipsychotics in the intensive care unit.. Crit Care Med..

[6] Oğlu GI (2016;). Potential Drug–Drug Interactions In A Medical Intensive Care Unit Of A University Hospital.. Turk J Med Sci..

[7] Hamidy YM,, Fauzia D. (2017;). Significant Drug Interactions Among Intensive Care Unit Patients.. Asian Journal of Pharmaceutical And Clinical Research..

[8] Bhaskaran J,, Johnson E, Population Trends In Substances Used In Deliberate Self-Poisoning Leading To Intensive Care Unit Admissions From 2000 To 2010.. J Clin Psychiatry.2015;.

[9] Ward M,, Schwartz A. (2013;). Challenges in pharmacologic management of the hospitalized patient with psychiatric comorbidity.. Journal of Hospital Medicine.

[10] American Thoracic Society. (1995;). Standards For The Diagnosis And Care Of Patients With Chronic Obstructive Pulmonary Disease.. Am J Respir Crit Care Med..

[11] Gacouin A,, Maamar A, (2017;). Patients With Preexisting Psychiatric Disorders Admitted To ICU: A Descriptive And Retrospective Cohort Study.. Ann. Intensive Care.

[12] Wunsch H,, Christiansen CF, (2014;). Psychiatric Diagnoses and Psychoactive Medication Use Among Nonsurgical Critically Ill Patients Receiving Mechanical Ventilation.. JAMA..

[13] Shafiekhani M,, Mirjalili M, (2018;). Psychotropic Drug Therapy In Patients. Therapeutics and Clinical Risk Management.

[14] Kutscher EC,, Alexander B. A Review of Drug Interactions With Psychiatric Medicines for the Pharmacy Practitioner.. Journal of Pharmacy Practice 2007;.

[15] Beach SR,, Celano CM (2013;). QTc Prolongation, Torsades de Pointes, and Psychotropic Medications.. Psychosomatics.

[16] Strain JJ,, Caliendo G (1999;). Cardiac Drug and Psychotropic Drug Interactions: Significance and Recommendations.. Gen Hosp Psychiatry..

